# Synthesis and Antitumor Activity of Diterpenylhydroquinone Derivatives of Natural *Ent*-Labdanes

**DOI:** 10.3390/molecules15096502

**Published:** 2010-09-17

**Authors:** Luis Espinoza Catalán, Evelyn Baeza Maturana, Karen Catalán Marín, Mauricio Osorio Olivares, Héctor Carrasco Altamirano, Mauricio Cuellar Fritis, Joan Villena García

**Affiliations:** 1 Departamento de Química, Universidad Técnica Federico Santa María, Av. España N 1680, Valparaíso, Chile; 2 Departamento de Ciencias Químicas, Universidad Andrés Bello, Campus Viña del Mar, Los Fresnos N 52, Viña del Mar, Chile; 3 Facultad de Farmacia, Universidad de Valparaíso, Av. Gran Bretaña N 1093, Valparaíso, Chile; 4 Facultad de Medicina, Universidad de Valparaíso, Av. Hontaneda N 2664, Centro Regional de Estudios en Alimentos Saludables (CREAS), Valparaíso, Chile

**Keywords:** diterpenylhydroquinones, *ent*-labdanes, antitumoral activity

## Abstract

Two new compounds 2β-acetoxy-15-phenyl-(22,25-acetoxy)-*ent*-labda-8(17), 13(*E*)-diene (**9**) and 2β-hydroxy-15-phenyl-(22,24,26-trimethoxy)-*ent*-labda-8(17),13(*E*)-diene (**10**) have been prepared by an Electrophilic Aromatic Substitution (*EAS*) reaction between diterpenyl allylic alcohols and 1,4-hydroquinone or 1,3,5-trimethoxybenzene using BF_3_^.^Et_2_O as a catalyst. These compounds, along with a series of natural *ent*-labdanes **3**-**8**, have been evaluated for their *in vitro* cytotoxic activities against cultured human cancer cells of PC-3 and DU-145 human prostate cancer, MCF-7 and MDA-MB-231 breast carcinoma and dermal human fibroblasts (DHF). Some compounds displayed inhibition at µM IC_50 _values.

## 1. Introduction

**Figure 1:**
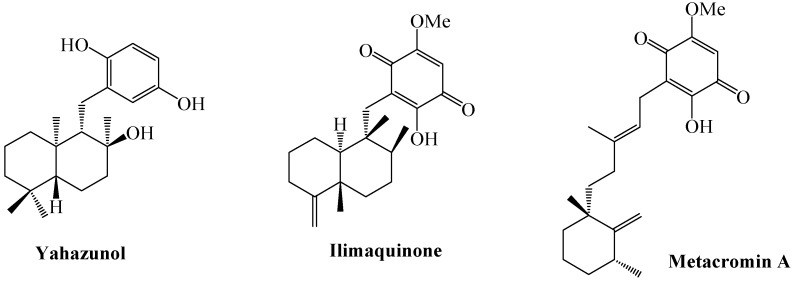
Some examples of natural sesquiterpenylquinones.

Terpenylquinones constitute an interesting group of marine natural products, for which a wide variety of biological activities have been described, including anti-inflammatory, antifungal, anti-HIV and most frequently, antineoplastic properties [1,2]. Their cytotoxic activities has been justified by their ability undergo redox cycles and the generation of reactive oxygen species, which could damage tumour cells [3,4]. These compounds are generally characterized for having a sesquiterpene skeleton attached to a (hydro)quinone moiety (Figure 1).

Terpenylquinones are isolated from natural sources in very low yield, and for that reason during the last few decades, considerable research effort has been focused on obtaining these compounds by synthesis. The most recurrent strategies used for synthesizing these compounds involve, as a first step, the separate preparation of the appropriate terpenyl fragments and aromatic nucleus. The crucial step is the attachment of the aromatic synthon to the terpenyl skeleton. There are many publications that report different methods for accomplishing these coupling reactions in the synthesis of sesquiterpenyl-quinones [5,6,7,8,9,10,11,12]. On the other hand, isolation of natural diterpenylquinones has not been reported and only one work has reported the synthesis of diterpenylnaphthoquinones, which showed an important activity in the inhibition of the growth of cancerigenous cells [1,2].

**Figure 2:**
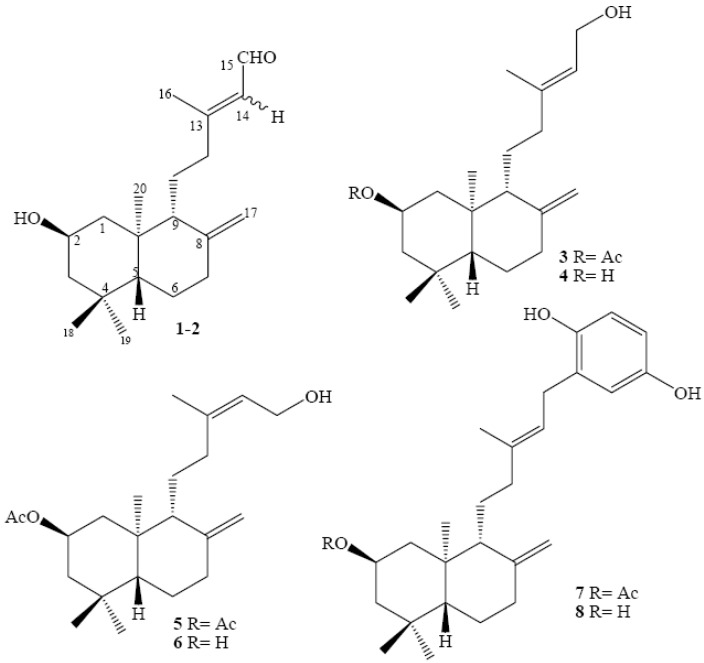
Series of natural *ent*-labdanes derivatives.

Recently we reported the synthesis and the structural determination of a series of natural *ent*-labdane derivatives **3**-**6** (Figure 2) and the diterpenylhydroquinones **7** and **8** (Figure 2) using Electrophilic Aromatic Substitution (*EAS*) reactions between an arene nucleus and an allylic alcohol as diterpenyl synthon [13].

**Figure 3:**
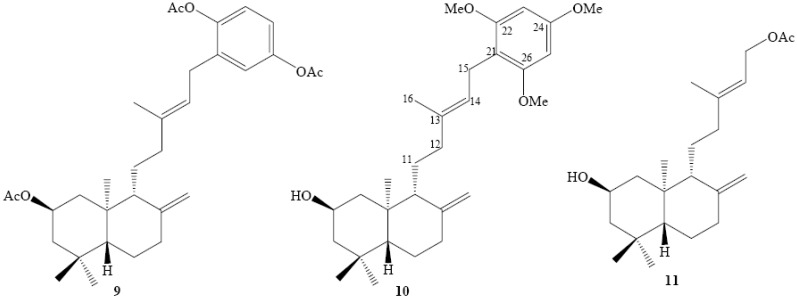
New diterpenylhydroquinone derivatives of natural *ent*-labdanes.

Using this synthetic protocol and as continuation of that research we describe in this paper the preparation of two new derivatives **9** and **10**. Additionally a new compound **11** derived from the natural *ent*-labdanes was obtained (Figure 3). Finally the new compounds **9**-**11** and the previously reported series of natural *ent*-labdanes derivatives **3**-**8** were evaluated *in vitro* against cultured human cancer cells in order to analyse the influence of the molecular structure on the antitumoral activity.

## 2. Results and Discussion

**Scheme 1:**
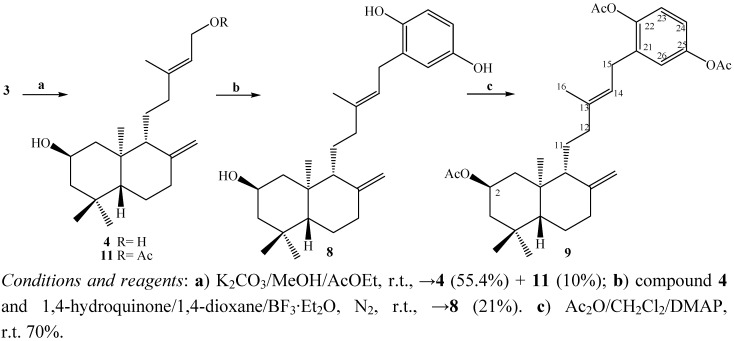
Synthesis of compound **9**.

Previously we reported the isolation and structural determination of the mixture of *ent*-labdanes **1**-**2** from *Calceolaria inamoena* and the preparation of derivatives **3** and **8 ** [13,14]. In the saponification reaction used for preparation of diterpenyl synthon **4**, a new compound **11** was obtained when acidic conditions were used, presumably due to a transesterification reaction and the presence of ethyl acetate in the workup procedure, nevertheless the target diol **4** could be obtained under partial neutralization conditions (Scheme 1).

The next step was the coupling reaction between compound **4** and 1,4-hydroquinone to give diterpenylhydroquinone **8**, using the synthetic protocol recently reported by us [13]. A subsequent acetylation reaction, under standard conditions, gave the new compound **9** (Scheme 1). The structural determination of compound **9** was accomplished by ^1^H, ^13^C, DEPT-135, gs-2D HSQC and gs-2D HMBC NMR techniques and by comparison with the spectral data of compound **8 ** [13]. 

**Scheme 2:**
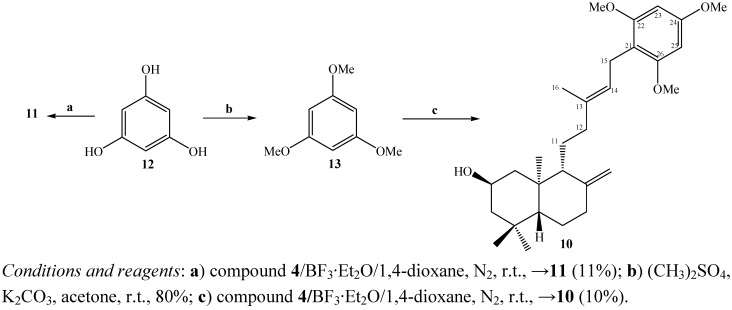
Synthesis of compound **10**.

On the other hand, the reaction between the diol **4** and 1,3,5-trihydroxybenzene (**12**) was not observed and compound **11** was unexpectedly obtained again, presumably for the same reasons given above (Scheme 2). The structure of the new natural *ent*-labdane derivative **11** was established by comparison with the previously reported spectral data of compound **4** [13].

**Figure 4:**
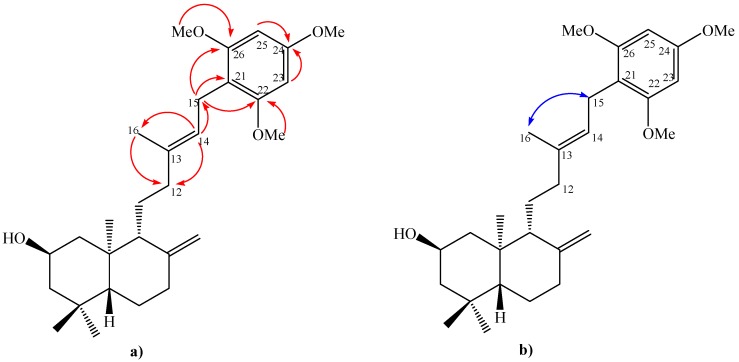
Structure of compound 10. (**a**) HMBC correlations; (**b**) NOE correlations.

The coupling reaction between the diol **4** and 1,3,5-trimethoxybenzene (**13, **obtained from **12**) rendered the new diterpenylhydroquinone **10** (Scheme 2). The ^1^H-NMR spectrum of compound **10** showed the existence of three methoxyl groups at δ 3.80 (s, 3H, CH_3_O); 3.79 (s, 3H, CH_3_O); 3.78 (s, 3H, CH_3_O) ppm and the presence of two aromatic hydrogens at δ 6.13 ppm (s, 2H, H-23 and H-25), furthermore in the ^13^C-NMR spectrum the presence of six aromatic carbons and three methoxyl groups at δ 55.7 (CH_3_O), 55.7 (CH_3_O), 55.3 (CH_3_O) ppm were also observed. On the other hand, the point of coupling between the diterpenyl fragment and the aromatic nuclei was confirmed by the presence of the signal at 3.25 ppm (dd, *J* = 10 and 7.5 Hz, 2H) assigned to the H-15 hydrogens, which was correlated (by 2D HSQC) with the carbon atom at δ 21.7 ppm (C-15) and corroborated by the 2D HMBC ^3^*J* heteronuclear correlations between H-15 and the carbon signals at δ 158.6 (C-22), 158.6 (C-26) ppm and ^2^*J* heteronuclear correlations with δ 111.1 (C-21) ppm (Figure 4a).

The *E*-geometry of the trisubstituted C13-C14 double bond was deduced from gs-sel-^1^H 1D-NOESY experiments, where H-15 showed a long range interaction with the Me-16 group (1.73 ppm) (Figure 4b).

### Bioactivity

The antitumor cytotoxicity of the compounds was evaluated *in vitro* against three different cancer cell lines: PC-3 and DU-145 human prostate cancer, MCF-7 and MDA-MB-231 breast carcinoma and one non-tumoral cell line, dermal human fibroblasts (DHF). A conventional colorimetric assay was set up to estimate the IC_50_ values, which represent the concentration of a drug that is required for 50% inhibition *in vitro* after 72 h of continuous exposure to the test compounds. Four serial dilutions (from 12.5 to 100 µM) for each sample were evaluated in triplicate. The results obtained from these assays are shown in Table 1 and the following general observations can be made as a result: the natural *ent*-labdanes derivatives **3**-**5** (IC_50_ > 100) were the least cytotoxic of the series. For cell line PC-3 compounds **7**-**8** were the most active, with IC_50_ values ranging between 21.45 µM and 28.24 µM, suggesting that the presence of the hydroquinone moiety significantly increase the bioactivity. The highest cytotoxicity values were observed for the diterpenylhydroquinone compounds **7** (IC_50_ 21.45 µM, prostate cancer PC-3), **8** (IC_50_ 28.24 µM, prostate cancer MCF-7) and **9** (IC_50_ 33.83 µM, breast cancer MDA-MB-231). Additionally, the cytotoxicity of the compounds **7**-**9** in fibroblast cells is lower than in the cancer cell lines under study, indicating that the compounds have higher selectivity for cancer cells.

**Table 1:** Cytotoxicity (IC_50_ µM) of natural *ent*-labdane derivatives **3**-**11.**

Compound	DU-145	MCF-7	MDA-MB-231	PC-3	DHF
3	> 100	s	> 100	> 100	> 100
4	> 100	90,56	> 100	> 100	> 100
5	> 100	> 100	> 100	> 100	> 100
6	96,19	74,94	95,98	>100	>100
7	44,61	23,9	75,86	21,45	>100
8	37,58	33,95	61,78	28,24	>100
9	62,91	41,11	33,83	64,29	72,30
10	> 100	45,62	85,93	91,17	> 100
11	> 100	86,92	> 100	35,58	> 100

## 3. Conclusions

As a conclusion, we have described the synthesis and structural determination of two new diterpenylhydroquinones **9** and **10 **from natural *ent*-labdane derivatives. These compounds were obtained by Electrophilic Aromatic Substitution (EAS) coupling reactions between primary allylic alcohol *ent*-labdane derivatives with1,4-hydroquinone and 1,3,5-trimethoxybenzene. Furthermore, a new co-compound **11** derived from a natural *ent*-labdane was obtained. The new compounds **9 **and **10** showed cytotoxic bioactivity against cell lines MCF-7 and MDA-MB-231 breast carcinoma. The natural *ent*-labdane derivatives **3**-**6** and **11** did not affect the bioactivity of the cells lines studied, however the diterpenylhydroquinones derivatives **7**-**10** showed IC_50_ values with significant inhibitory activity, ranging in the µM level, due to the presence of a hydroquinone moiety. Moreover, compounds **7**-**9** showed some selectivity for cancer cells *versus* fibroblast cells, which could be a way to access to compounds potentially less toxicity to normal human cells.

## 4. Experimental

### 4.1. General

All purchased chemical reagents (Merck or Aldrich) were of the highest commercial available purity and were used without previous purification. IR spectra were recorded as thin films on a Nicolet Impact 420 spectrometer and frequencies are reported in cm^−1^. Optical rotations were measured with a sodium lamp (λ = 589 nm, D line) on a Perkin Elmer 241 digital polarimeter equipped with 1 dm cells at the temperature indicated in each case. Low resolution mass spectra were recorded on a Shimadzu QP-2000 spectrometer at 70eV ionising voltage and are given as *m/z* (% rel. int.). ^1^H, ^13^C (DEPT 135), sel. 1D ^1^H NOESY, 2D HSQC and 2D HMBC spectra were recorded in CDCl_3_ solutions on a Bruker Avance 400 Digital NMR spectrometer, operating at 400.1 MHz for ^1^H and 100.6 MHz for ^13^C and are referenced to the residual peaks of CHCl_3_ at δ 7.26 ppm and δ 77.0 ppm for ^1^H and ^13^C, respectively. Chemical shifts are reported in δ ppm and coupling constants (*J*) are given in Hz. Silica gel (Merck 200-300 mesh) was used for column chromatography and HF-254 silica gel plates for TLC. TLC spots were detected by heating after spraying with 25% H_2_SO_4_ in H_2_O.

### 4.2. Starting Materials

Natural *ent*-labdanes **1-2** were isolated from *Calceolaria inamoena* and transformed into diterpenyl allylic alcohol derivative **4** by a reported procedure [13,14].

### 4.3. General Procedure for EAS: Synthesis of Compounds ***8, 10***

BF_3_^.^Et_2_O (0.23 g, 1.6 mmol) was gradually added at room temperature to a solution of 1,4-hydro-quinone (0.30 g, 2.27 mmol) or 1,3,5-trimethoxybenzene (0.30 g, 1.79 mmol) and compound **3** (0.50 g, 1.6 mmol) in freshly distilled 1,4-dioxane (30 mL). The mixture was stirred at room temperature under a nitrogen atmosphere for 24 h, when the completion of the reaction was verified by TLC. The mixture was poured onto crushed ice (about 30 g) and the organic layer was extracted with EtOAc (3 × 30 mL). The combined organic phase was washed with 5% NaHCO_3_ (30 mL), then with water (2 × 20 mL) and dried over anhydrous Na_2_SO_4_, filtered and evaporated. The crude residue was redissolved in CH_2_Cl_2_(5 mL) and chromatographed on silica gel with petroleum ether/EtOAc mixtures of increasing polarity (19.8:0.2→10.2:9.8) for **8** and (19.8:0.2→13.0:7.0) for **10**.

*2β-Acetoxy-15-phenyl-(22,25-acetoxy)-ent-labda-8(17),13(E)-diene* (**9**). To diterpenylhydro-quinone **8** (48.8 mg, 0.12 mmol) dissolved in dichloromethane (30 mL), acetic anhydride (0.54 g, 0.5 mL, 5.2 mmol) and dimethylaminopyridine (1.5 mg, 0.01 mmol) were added. The mixture was stirred at room temperature during 2 h. The organic phase was washed with water and after it was dried over anhydrous Na_2_SO_4_. The solution was evaporated to dryness to afford the crude reaction product, which gave 45 mg of compound **9** after column chromatography (eluent hexane/ethyl acetate 19.8:0.2→15.8:4.2), Yield 70%. Colorless viscous oil; [α]_D_^23^ = −18.1º (c 0.38, CHCl_3_); ^1^H-NMR: 7.02 (d, *J* = 9.5 Hz, 1H, H-23); 6.93 (m, 2H, H-24 and H-26); 5.17 (bt, *J* = 7.1 Hz, 1H, H-14); 5.01 (ddt, *J* = 12.0, 12.0 and 4.2 Hz, 1H, H-2); 4.86 (s, 1H, H-17b); 4.55 (s, 1H, H-17a); 3.22 (d, *J* = 7.1 Hz, 2H, H-15); 2.38 (ddd, *J* = 13.0, 4.9 and 2.2 Hz, 1H, H-7α); 2.30 (s, 3H, CH_3_CO_2_-C-22); 2.28 (s, 3H, CH_3_CO_2_-C-25); 2.15 (ddd, *J* = 14.2, 13.0 and 4.4 Hz, 1H, H-12b); 2.05 (m, 1H, H-1α); 2.03 (s, 3H, CH_3_CO_2_-C-2); 1.92 (ddd, *J* = 13.5, 13.0 and 4.9 Hz, 1H, H-7β); 1.83 (m, 1H, H-12a); 1.75 (m, 1H, H-3α); 1.71 (m, 1H, H-6α); 1.67 (s, 3H, H-16); 1.62 (bd, *J* = 11.0 Hz, 1H, H-9); 1.55 (m, 1H, H-11b); 1.46 (ddd, *J* = 12.2, 9.8 and 4.4 Hz, 1H, H-11a); 1.29 (dd, *J* = 13.0 and 3.9 Hz, 1H, H-6β); 1.24 (dd, *J* = 12.0 and 12.0 Hz, 1H, H-3β); 1.08 (dd, *J* = 13.0 and 2.5 Hz, 1H, H-5); 1.04 (dd, *J* = 12.0 and 12.0 Hz, 1H, H-1β); 0.93 (s, 3H, H-18); 0.88 (s, 3H, H-19); 0.76 (s, 3H, H-20); ^13^C-NMR: 44.1 (C-1), 69.3 (C-2), 46.8 (C-3), 34.9 (C-4), 54.9 (C-5), 23.9 (C-6), 37.9 (C-7), 147.3 (C-8), 55.9 (C-9), 40.9 (C-10), 22.1 (C-11), 38.3 (C-12), 137.8 (C-13), 120.8 (C-14), 28.6 (C-15), 16.2 (C-16), 107.3 (C-17), 33.5 (C-18), 22.4 (C-19), 15.2 (C-20), 134.9 (C-21), 146.2 (C-22), 122.9 (C-23), 119.9 (C-24), 148.2 (C-25), 122.7 (C-26), 170.6 (CH_3_COC-2), 169.3 (CH_3_COC-Ar), 169.2 (CH_3_COC-Ar), 21.5 (CH_3_CO_2_C-2), 21.1 (CH_3_CO_2_Ar-25), 20.8 (CH_3_CO_2_Ar-22); IR (cm^−1^): 2940, 1767, 1737, 1486, 1363, 1239, 1209, 1168, 1025; MS (*m/z*, %): M^+^ 524 (< 1%), 168 (9.5), 167 (100), 132 (6.3), 122 (4.5), 121 (4.3), 113 (25), 112 (14.9), 105 (5.5), 104 (15.2), 93 (4.1), 84 (7.7), 83 (13.8), 82 (2.3), 76 (7.3), 71 (37.3), 70 (31.7), 69 (8.5), 57 (45.5), 55 (17.4).

*2β-Hydroxy-15-phenyl-(22,24,26-trimethoxy)-ent-labda-8(17),13(E)-diene* (**10**)*.* colorless viscous oil, 70 mg, Yield 10%, [α]_D_^23^ = −6.2º (c 1.66, CHCl_3_); ^1^H-NMR: 6.13 (s, 2H, H-23 and H-25); 5.12(t, *J* = 7.2 Hz, 1H, H-14); 4.81 (s, 1H, H-17b); 4.53 (s, 1H, H-17a); 3.83 (ddt, *J* = 11.5, 11.5 and 4.2 Hz, 1H, H-2); 3.80 (s, 3H, CH_3_O);3.79 (s, 3H, CH_3_O); 3.78 (s, 3H, CH_3_O); 3.25 (dd, *J* = 10 and 7.5 Hz, 2H, H-15); 2.33 (ddd, *J* = 12.7, 4.4 and 2.2 Hz, 1H, H-7α); 2.17 (m, 1H, H-12b); 2.10 (m, 1H, H-1a); 1.84 (ddd, *J* = 13.5, 13.2 and 4.6 Hz, 1H, H-7β); 1.83 (m, 1H, H-12a); 1.75 (m, 1H, H-3α); 1.73 (s, 3H, H-16); 1.68 (m, 1H, H-6β); 1.57 (bd, *J* = 9.9 Hz, 1H, H-9); 1.55 (m, 2H, H-11a and H 11-b); 1.26 (m, 1H, H-6α); 1.09 (dd, *J* = 12.0 and 12.0 Hz, 1H, H-3β); 0.97 (dd, *J* = 12.6 and 2.6 Hz, 1H, H-5); 0.91 (s, 3H, H-18); 0.82 (s, 3H, H-19); 0.68 (s, 3H, H-20); ^13^C-NMR: 48.1 (C-1), 65.7 (C-2), 51.1 (C-3), 35.0 (C-4), 54.8 (C-5), 23.9 (C-6), 38.2 (C-7), 147.8 (C-8), 55.5 (C-9), 40.9 (C-10), 21.9 (C-11), 38.0 (C-12), 134.4 (C-13), 123.5 (C-14), 21.7 (C-15), 16.1 (C-16), 107.0 (C-17), 33.6 (C-18), 22.6 (C-19), 15.4 (C-20), 111.1 (C-21), 158.6 (C-22), 90.7 (C-23), 159.1 (C-24), 90.7 (C-25), 158.6 (C-26), 55.7 (CH_3_O), 55.7 (CH_3_O), 55.3 (CH_3_O); IR (cm^−1^): 2953, 2927, 1727, 1601, 1460, 1207, 1152, 1124, 1036, 750; MS (*m/z*, %): M^+^ 456 (< 1%), 429 (19), 357 (24), 356 (35), 355 (100), 281 (47), 221 (48), 207 (18), 147 (53), 73 (79).

*2β-Hydroxy-15-acetoxy-ent-labda-8(17),13(E)-diene* (**11**). To compound **3** (0.22 g, 0.63 mmol) dissolved in methanol (70 mL), potassium carbonate (0.1 g, 0.72 mmol) was added. The mixture was stirred at room temperature during 2 h. The solution was evaporated to dryness. Dilution with ethyl acetate was followed by washing with HCl 5% and organic phase was dried over anhydrous Na_2_SO_4_. The solution was evaporated to dryness to afford the crude reaction product, which gave 22 mg of compound **11** after column chromatography (eluent hexane/ethyl acetate 19.8:0.2→16.0:4.0), Yield 10%. colorless viscous oil, [α]_D_^23^ = −27.6º (c 0.88, CHCl_3_); ^1^H-NMR: 5.35 (t, *J* = 7.3, 1H, H-14); 4.90 (s, 1H, H-17b); 4.58 (s, 1H, H-17a); 4.50 (dd, *J* = 7.0, and 3.0 Hz, 2H, H-15); 3.87 (ddt, *J* = 4.2, 11.5 and 11.4 Hz, 1H, H-2); 2.4 (ddd, *J* = 12.8, 4.1 and 2.4 Hz, 1H, H-7α); 2.17 (m, 1H, H-12b); 2.10 (m, 1H, H-1α); 2.04 (s, 3H, CH_3_CO_2_-C-15); 1.96 (ddd, *J* = 13.0, 13.0 and 5.0 Hz, 1H, H-7β); 1.83 (m, 1H, H-12a); 1.78 (dd, *J* = 2.3 and 4.1, 1H, H-3α); 1.75 (s, 3H, H-16); 1.72 (m, 1H, H-6β); 1.61 (bd, *J* = 10.4 Hz, 1H, H-9); 1.47 (m, 2H, H-11a and H-11b); 1.29 (dd, *J* = 12.9 and 4.2 Hz, 1H, H-6α); 1.15 (dd, *J* = 12.0 and 12.0 Hz, 1H, H-3β); 1.07 (dd, *J* = 12.6 and 2.6 Hz, 1H, H-5); 0.95 (dd, *J* = 11.5 and 11.5 Hz, 1H, H-1β); 0.93 (s, 3H, H-18); 0.84 (s, 3H, H-19); 0.71 (s, 3H, H-20); ^13^C-NMR: 48.2 (C-1), 65.7 (C-2), 51.1 (C-3), 33.7 (C-4), 54.9 (C-5), 23.9 (C-6), 38.3 (C-7), 147.6 (C-8), 56.1 (C-9), 41.0 (C-10), 21.8(C-11), 38.0 (C-12), 142.8 (C-13), 118.1 (C-14), 61.4 (C-15), 16.5 (C-16), 107.2 (C-17), 35.0 (C-18), 22.6 (C-19), 15.4 (C-20), 21.1 (CH_3_CO_2_-C-15), 171.2 (CO_2_-C-15); IR (cm^−1^): 2934, 2856, 1725, 1461, 1441, 1366, 1259, 1235, 1029, 737; MS (*m/z*, %): M^+^ 348.52 (< 1%), 318 (25), 175 (34), 135 (100), 121 (28), 119 (25), 107 (35), 105 (25), 95 (28), 93 (34), 81 (30), 69 (34), 57 (25).

### 4.4. Bioactivity: Cell Growth Inhibition Assay

The colorimetric assay using sulforhodamine B (SRB) was realized following an adaptation of the described method by Skehan *et al*. [15,16]. Cells were seeded in 96-well microtiter plates, at5 × 10^3^ cells per well in aliquots of 100 µL of DMEM/F-12 medium, and they were allowed to attach to the plate surface by growing in drug-free medium for 18 h. Afterward, compounds samples were added in aliquots (dissolved in EtOH/H_2_O) to achieve a final concentration of 100, 50, 25 12.5 µM. After 72 h exposure, the *in vitro* cytotoxicity was measured by the SRB dye assay. Cells were fixed by adding cold 50% (wt/vol) trichloroacetic acid (TCA, 25 µL) and incubating for 60 min at 4ºC. Plates were washed with deionized water and dried; SRB solution (0.1% wt/vol in 1% acetic acid, 50 µL) was added to each microtiter well and incubated for 30 min at room temperature. Unbound SRB is removed by washing with 1% acetic acid. Plates were air-dried and bound stain was solubilized with Tris base (100 µL, 10 mM). Optical densities were read on an automated spectrophotometer plate reader at a single wavelength of 540 nm. Values shown are the % viability *vs.* ctrl + SD, n = four independent experiments in triplicate.
